# The Role of Children’s Dietary Pattern and Physical Activity in the Association Between Breastfeeding and BMI at Age 5: The GECKO Drenthe Cohort

**DOI:** 10.1007/s10995-020-03063-6

**Published:** 2020-11-30

**Authors:** Petra Corianne Vinke, Carolien Tigelaar, Leanne Karen Küpers, Eva Corpeleijn

**Affiliations:** 1 Department of Epidemiology, University Medical Center Groningen, University of Groningen, P.O. Box 30 001, Groningen, 9700 RB The Netherlands; 2 Division of Human Nutrition and Health, Wageningen University & Research, PO Box 8129, Wageningen, 6700 EV The Netherlands

**Keywords:** Breast feeding, Childhood obesity, Healthy diet, Physical activity, Health literacy

## Abstract

**Objectives:**

Breastfeeding is protective against childhood obesity, but the role of childhood lifestyle in this association is unclear. We investigated whether physical activity and dietary pattern at age 5 differed between breastfed and non-breastfed children, and how they relate to Body Mass Index (BMI) Z-scores.

**Methods:**

1477 children of the Dutch GECKO Drenthe birth cohort were included. At one month, children were categorized as breastfed (receiving breast milk exclusively or in combination with formula milk) or non-breastfed (receiving formula milk exclusively). At age 5, height and weight were objectively measured, physical activity was measured by ActiGraph GT3x and dietary patterns were assessed with a parent-reported food pattern questionnaire, assessing the consumption frequency of selected food items at seven occasions over the day.

**Results:**

Non-breastfed children had higher BMI Z-scores (0.36 ± 0.90 vs. 0.20 ± 0.80 SD, p = 0.002), more frequently consumed sugar-sweetened beverages (25.0 ± 10.5 vs. 22.5 ± 9.71 times per week, p < 0.001), and consumed relatively less whole-wheat or brown bread (p = 0.007). Differences in sugar-sweetened beverage consumption were most pronounced during main meals. Total fruit consumption, sedentary time and moderate-to-vigorous physical activity levels did not differ between the groups. Multivariable adjusted linear regression analyses showed that the differences in BMI-z score between non-breastfed and breastfed children were not explained by the differences in sugar-sweetened beverages or type of bread consumed.

**Conclusions:**

Infant breastfeeding itself is indicative of healthy dietary behaviors in early life, and is also more likely to be followed by a favorable dietary pattern at toddler age. However, the differences in dietary habits between breastfed and non-breastfed children did not explain the difference in BMI Z-score at the age of 5.

**Electronic supplementary material:**

The online version of this article (10.1007/s10995-020-03063-6) contains supplementary material, which is available to authorized users.

## Significance

*What is already known on this subject? *Breastfeeding is known to be associated with lower overweight and obesity risk, but little is known about the role of children’s dietary pattern and physical activity in this association. *What this study adds? *At age 5, the dietary pattern of breastfed children is characterized by healthier behaviours regarding sugar-sweetened beverage consumption, whole grain bread consumption and snacking. Although breastfed children have a lower BMI at age 5 than non-breastfed children, this difference was not explained by differences in dietary patterns. Since dietary behaviours in early childhood are largely dependent on parental choices, nutrition education for parents may be key to improve dietary patterns in children.

## Introduction

The rising worldwide prevalence of childhood obesity is one of the main challenges of the twenty-first century (Ng et al. 2014). Similar to adult obesity, childhood obesity is associated with elevated blood pressure, hyperlipidemia and increased risk for type 2 diabetes (Deckelbaum and Williams 2001; Gurnani et al. 2015). Moreover, childhood obesity is a strong predictor of obesity in adulthood (Deckelbaum and Williams 2001; Goran 2001; Nader et al. 2006).

The period from conception to two years of age is known as the “*first thousand days*” (Woo Baidal et al. 2016), in which maternal smoking during pregnancy, higher maternal pre-pregnancy body mass index (BMI) and higher infant birth weight influence the risk of childhood weight gain and obesity (Han et al. 2010; Küpers et al. 2015; Rayfield and Plugge 2017; Reilly et al. 2005; Woo Baidal et al. 2016). An important lifestyle factor in the first thousand days is breastfeeding. A meta-analysis regarding the long-term consequences of breastfeeding calculated a 26% lower overweight and obesity risk for breastfed compared to non-breastfed children in studies including children between the age of 1 and 9. For studies in adolescents, aged 10–19, this was 37%, and for studies in adults, aged ≥ 20, this was 12%. In this meta-analysis, subjects were classified as either breastfed or non-breastfed according to the criteria used in each study, which included various cut-offs within the first year after birth (Horta et al. 2015). The age-stratified results of this meta-analysis illustrate that the consequences of this early life factor are noticeable throughout an individual’s life course. Although most studies investigating the association between breastfeeding and childhood obesity take into account potential confounding factors such as birth weight, maternal BMI and socioeconomic status (SES) (Yan et al. 2014), little is known about the role of children’s lifestyle in this association.

Parents who choose to breastfeed their child are known to have a higher SES than non-breastfeeding parents (Amir and Donath 2008; Flacking et al. 2007). At the same time, higher SES is found to be associated with a healthier lifestyle in terms of nutrition (Darmon and Drewnowski 2008), smoking (Hiscock et al. 2012) and physical activity (Trost et al. 2002). It may therefore be hypothesized that high-SES parents who choose to breastfeed have a healthier lifestyle in general. As a consequence, this may also result in more optimal dietary patterns and physical activity levels among their children, rendering them less susceptible to overweight or obesity during childhood.

We aimed to compare children who received any breastmilk to those who were exclusively formula fed at the age of 1 month with regard to their physical activity levels and dietary patterns (covering food intake and timing of consumption) at the age of 5. Furthermore, we examined to what extent the beneficial association between breastfeeding and BMI was related to differences in these lifestyle components.

## Methods

### GECKO Drenthe Birth Cohort

The GECKO Drenthe birth cohort is a Dutch population-based birth cohort of children born in 2006 and 2007 that has been designed to study the determinants and development of childhood overweight. Further details regarding the study design, recruitment and study procedures have been published elsewhere (L’Abée et al. 2008). Children with a birth weight < 2500 g and/or a gestational age < 37 weeks were excluded. Additionally, children for whom data on breastfeeding at 1 month, or data on both diet and physical activity was missing, were excluded from the study (Figure S1). Written informed consent was obtained from all parents. The study was conducted in accordance with the Declaration of Helsinki and was approved by the Medical Ethics Committee of the University Medical Center Groningen. The cohort is registered at www.birthcohorts.net.

### Anthropometric Measurements

At the age of 5, anthropometric measurements were performed by trained staff of the Community Health Service in the province of Drenthe. Body weight was measured using an electronic scale with digital reading to the nearest 0.1 kg. Height was assessed to the nearest 0.1 cm. From weight and height, BMI-for-age Z-scores (BMIz) were calculated with Growth Analyser software, version 3.5 (DutchGrowthFoundation 2011), based on Dutch growth references from 1997 (Fredriks et al. 2000).

### Infant Feeding

To reflect parental motivation and persistence, rather than ever or never receiving breast milk, the distinction between breastfed and non-breastfed children was made at the age of 1 month. At this age, parents were asked in a questionnaire what type of milk their baby currently received (breast milk, pumped breast milk or formula milk–choosing more than one answer was possible). As this study focuses on behavioural aspects of lifestyle rather than biological aspects, children who received breast milk exclusively or breast milk in combination with formula milk or pumped breast milk were categorized as breastfed. Children were categorized as non-breastfed when they received formula milk exclusively. An alternative classification in which only children who were exclusively breastfed at the age of 1 month were categorised as breastfed was used in a sensitivity analysis.

### Physical Activity

Physical activity (PA) at age 5/6 was measured in counts per minute (cpm) using the ActiGraph GT3X (ActiGraph, Pensacola, FL), a reliable and valid accelerometer to measure PA volume and intensity in young children (Hänggi et al. 2013; Sirard et al. 2005). Participants were instructed to wear the ActiGraph on the iliac crest on the right hip, for four days, of which at least one weekend day, during all waking hours except while bathing or swimming (Penpraze et al. 2006; Sigmund et al. 2007). To be included in the analysis, the accelerometer had to be worn for at least 600 min/day for at least 3 days, regardless of whether these were week or weekend days. Non-wearing time of the ActiGraph was classified as a minimum length of 90 min without any observed counts (Choi et al.^2012^). Collected data were analysed in 15-s epochs. Data were collected using a frequency of 30 Hz (McClain et al. 2008). All children with wearing time ≥ 840 min/day (14 h/day) were checked manually to exclude sleeping time. Thresholds for vector magnitude recommended by Butte et al. were used to identify the amount of sedentary time [< 819 counts per minute (cpm)], and moderate-to-vigorous PA (MVPA) (≥ 3908 counts per minute) (Butte et al. 2014).

### Dietary Habits

Data regarding dietary habits was collected using a detailed parent-reported food pattern questionnaire (241 items), filled in at the child’s age of 5. The questionnaire was based on the questionnaire used for ChecKid, a study focussing on the behaviour, health and living circumstances of children living in the Netherlands (de Jong et al. 2013). This questionnaire differs from more commonly used food frequency questionnaires, in the sense that it does not ask for the overall daily frequency of consumption of selected food items. Instead, it asks for the frequency of consumption of selected food items at seven moments of the day (breakfast, in the morning, lunch at school, lunch at home, in the afternoon, dinner, and in the evening). The answer possibilities were (i) never, (ii) 0–1 times a week, (iii) 2–3 times a week, (iv) 4–5 times a week, or (v) 6–7 times a week. For analyses, these answers were recoded to continuous values (frequency per week) using the midpoint value of the categorical answer. Data were checked for face validity, including completeness, consistency and realistic values.

Based on the 2015 Dutch Dietary Guidelines (Kromhout et al. 2016) and data availability, we chose to particularly study three dietary components relevant to weight maintenance: type of bread (Maki et al. 2019), sugar sweetened beverages (SSB) and fruit (Keller et al. 2015; Schlesinger et al. 2019). Except for SSB, this evidence mostly comes from studies in adults, but we have no reason to believe these food groups would not be relevant for childhood weight maintenance. Type of bread was chosen to represent the choice for refined or whole grain products, as in the Netherlands bread accounts for 65% of total cereal product consumption among children (Dutch National Food Consumption Survey 2010).

To score the type of bread, five categories to represent the proportion of white, brown (made with a mixture of whole wheat and white flour) and whole-wheat bread (all flour is whole-wheat flour) were defined 1*: white* (> 75%), 2: *brown* + *whole-wheat* + *white* (> 75%), 3: *brown* (> 75%), 4: *brown* + *whole-wheat* (> 75%), and 5: *whole-wheat* (> 75%). For example, if the percentage of white bread was > 75%, this means that at least 75% of slices of bread was ususally white bread, and that this was scored as 1. A higher score represents a higher consumption of whole-wheat bread relative to brown, or brown bread relative to white bread, and was therefore considered healthier. For fruit and SSB, intake was expressed as the frequency of consumption per week. This represented the sum of frequencies for the seven moments of the day that were specified earlier. Sweetened dairy drinks (including sweetened milk or yoghurt drinks and chocolate milk), sweetened lemonade, soda, fruit juice and tea with added sugar were classified as SSB. Portion sizes were not specified in the questionnaire.

For each moment of the day as well as for the whole day, the percentage of children consuming items from the three food groups of interest was determined. The proportions “consumers vs. non-consumers” were compared between breastfed and non-breastfed children (i.e. does the percentage of breastfed and non-breastfed children that ever consume fruit during breakfast differ?). Then, the mean breadscore, and SSB and fruit consumption frequency among the consumers was calculated. The consumer means of breastfed and non-breastfed children were compared as well (i.e. within the children that ever consume fruit during breakfast, does the frequency differ between breastfed and non-breastfed children?).

### Parental Characteristics

Data on parental characteristics (parental date of birth, parental BMI before pregnancy, smoking during pregnancy, highest net household income (≤ €1150/month, €1151-€3050/month, €3051-€3500/month, > 3500/month), parental education level [high ((applied) university) vs. low/middle (other))] was collected through self-reported questionnaires upon enrolment during pregnancy. Maternal smoking during pregnancy was cross-checked with records from the midwife.

### Statistical Analysis

The five main lifestyle factors of interest (sedentary time, MVPA, type of bread, SSB, fruit) were normally distributed, but since other factors were not, for consistency in descriptive statistics, Mann–Whitney U tests were used for continuous and ordinal variables and the Fisher’s exact for categorical variables. A Spearman’s rank correlation test was performed to test the correlation between food items at different moments of the day.

The association of breastfeeding with the five selected lifestyle components was investigated in linear regression models. It was checked whether assumptions of linear regression (linearity of association, normality of variables and homoscedasticity of residuals) were met. The first model was adjusted for child characteristics that were a priori identified as potential confounders (birth weight, gestational age, gender and ethnicity). In an additive manner, the second model was further adjusted for parental lifestyle factors (parental BMI, smoking during pregnancy) that could be characteristic of both breastfeeding habits as well as the child eating pattern. The third model was additionally adjusted for socioeconomic factors (household income, parental age at birth, parental educational level) to investigate to which extent the differences in breastfeeding and lifestyle habits could be linked to differences in socio-economic status.

To examine the extent to which the beneficial association between breastfeeding and BMI was related to differences in lifestyle components of the child, the association between breastfeeding and BMIz was investigated with an additional multivariate linear regression with BMIz as the dependent variable. This model included an additional step preceding the inclusion of parental lifestyle factors and socioeconomic factors, in which the child’s lifestyle characteristics were added that were significant in the previous analyses with basic confounder adjustment.

For 12.9% of the children, data on household income was missing or parents indicated that they were not willing to report their income. Therefore, household income was imputed with unstandardized predicted values derived from a linear regression with parental education level, socio-economic deprivation score based on postal code and maternal age as independent factors. In all models, variables were checked for collinearity. Paternal age was highly correlated with maternal age (r = 0.676, p < 0.000), wherefore it was not included in the analyses. An interaction term for gender and breastfeeding was added to all basic models to test whether gender was an effect modifier of the associations.

Analyses were performed in IBM SPSS 23 for Windows (SPSS, Chicago Illinois, USA). The level of significance was set to 5%.

## Results

Of the 1477 children who met the inclusion criteria (Figure S1), 63.4% received breastfeeding at the age of 1 month. Characteristics of the participants are shown in Table 1. As expected, non-breastfed children had significantly higher BMI z-scores and more overweight at the age of 5. Parents of non-breastfed children differed in age, income and education, and also in lifestyle characteristics, i.e. they had a higher BMI, and mothers who did not breastfeed their child smoked more frequently during pregnancy.

**Table 1.** Descriptive data of study population (N = 1477)

	N	Breastfed children	N	Non-breastfed children	P
Child characteristics					
Female gender, n (%)	936	489 (52.2%)	541	244 (45.1%)	**0.009**
Birth-weight (kg)	909	3.64 ± 0.47	530	3.59 ± 0.50	0.141
Gestational age (weeks)	931	40.1 ± 1.2	537	39.9 ± 1.3	** < 0.001**
Ethnicity (%)	873		504		**0.009**
Dutch		810 (92.8%)		485 (96.2%)	
Non-Dutch		63 (7.2%)		19 (3.8%)	
Age at PA measurement (years)	622	5.64 ± 0.79	284	5.67 ± 0.77	0.661
Age at dietary measurement (years)	874	5.81 ± 0.33	516	5.85 ± 0.33	0.118
BMI z-score at 5 years	836	0.20 ± 0.80	491	0.36 ± 0.90	**0.002**
Overweight at 5 years, n (%)	836	109 (13.0%)	491	92 (18.7%)	**0.007**
Parental characteristics					
Age father at birth (years)	890	33.89 ± 4.88	514	33.52 ± 4.44	0.261
Age mother at birth (years)	932	31.19 ± 4.22	540	30.80 ± 4.13	**0.050**
BMI father before pregnancy	862	25.32 ± 3.15	499	25.92 ± 3.55	**0.002**
BMI mother before pregnancy	889	24.14 ± 4.33	513	26.17 ± 5.22	** < 0.001**
Household income, n (%)	834		463		** < 0.001**
≤ €1150/month		25 (3.0%)		16 (3.5%)	
€1151–3050/month		512 (61.4%)		342 (73.9%)	
€3051–3500/month		171 (20.5%)		72 (15.6%)	
€3501 or more/month		126 (15.1%)		33 (7.1%)	
Educational level father, n (%)	886		511		** < 0.001**
Low/middle		530 (59.8%)		396 (77.5%)	
High [(applied) university]		356 (40.2)		115 (22.5%)	
Educational level mother, n (%)	907		524		** < 0.001**
Low/middle		462 (50.9%)		398 (76%)	
High [(applied) university]		445 (49.1%)		126 (24%)	
Smoking during pregnancy, n(%)	926		534		**0.001**
Yes		92 (9.9%)		85 (15.9%)	
No		834 (90.1%)		449 (84.1%)	

Bold: p < 0.05, N_breastfed_ = 936, N_non-breastfed_ = 541

Results are shown as mean ± SD or n (%)

### Physical Activity

Valid ActiGraph data was available for 622 breastfed and 284 non-breastfed children. Median daily sedentary time (373 min vs 368, p = 0.497) and moderate to vigorous physical activity (62 min/d vs. 62 min/d, p = 0.78) did not significantly differ between breastfed and non-breastfed children, respectively.

### Dietary Patterns

Valid food pattern questionnaire data at age 5 was available for 774 breastfed and 466 non-breastfed children. At breakfast, during lunch at home and in total, breastfed children had a higher bread score than non-breastfed children, indicating a relatively higher consumption of whole-wheat and brown bread versus white bread (Table 2). More detailed information on the types of bread consumed per eating occasion can be found in Online Resources (Table S1**)**.

**Table 2:** Comparison of breastfed and non-breastfed children with regard to consumer percentages and consumer means for type of bread, SSB and fruit intake

	Breastfed		Non-Breastfed			P-value *Consumer percentage*	P-value *Consumer intake*
	N_consumer_ (%)	Consumer intake	N_consumer_ (%)	Consumer intake			
Bread score							
Breakfast	733 (94.7%)	3.15 ± 1.08	433 (92.9%)	2.97 ± 1.05		0.216	**0.007**
Lunch at school	475 (61.4%)	3.30 ± 1.14	257 (55.2%)	3.21 ± 1.11		**0.032**	0.191
Lunch at home	741 (95.7%)	3.15 ± 1.14	453 (97.2%)	3.02 ± 1.11		0.216	**0.050**
Dinner	93 (12.0%)	3.05 ± 1.27	57 (12.2%)	2.75 ± 1.14		0.928	0.149
Total	771 (99.6%)	3.18 ± 1.09	465 (99.8%)	3.01 ± 1.07		1.000	**0.007**
Sugar-sweetened beverage consumption frequency							
Breakfast	520 (67.2%)	4.76 ± 2.55	352 (75.5%)		4.97 ± 2.50	**0.002**	0.302
In the morning	741 (95.7%)	5.89 ± 1.60	449 (96.4%)		5.83 ± 1.90	0.657	0.301
Lunch at school	342 (44.2%)	1.97 ± 1.48	214 (45.9%)		2.04 ± 1.50	0.556	0.523
Lunch at home	545 (70.4%)	4.32 ± 2.79	365 (78.3%)		4.91 ± 2.60	**0.002**	** < 0.001**
In the afternoon	750 (96.9%)	6.24 ± 2.09	453 (97.2%)		6.53 ± 2.59	0.864	0.105
Dinner	319 (41.2%)	4.13 ± 2.51	193 (41.4%)		4.36 ± 2.58	0.953	0.405
In the evening	354 (45.7%)	4.06 ± 2.55	271 (58.2%)		4.49 ± 2.89	** < 0.001**	0.221
Total	770 (99.5%)	22.5 ± 9.71	464 (99.6%)		25.0 ± 10.5	1.000	** < 0.001**
Fruit consumption frequency							
Breakfast	128 (16.5%)	2.38 ± 1.97	78 (16.7%)		2.71 ± 2.10	0.937	0.276
In the morning	721 (93.2%)	4.87 ± 1.44	422 (90.6%)		4.73 ± 1.47	0.103	0.091
Lunch at school	123 (15.9%)	1.41 ± 1.23	72 (15.5%)		1.64 ± 1.20	0.872	0.137
Lunch at home	162 (20.9%)	3.15 ± 2.17	98 (21.0%)		3.77 ± 1.98	1.000	**0.023**
In the afternoon	447 (57.8%)	3.19 ± 1.67	223 (47.9%)		2.99 ± 1.52	**0.001**	0.168
Dinner	148 (19.1%)	2.31 ± 1.74	57 (12.2%)		2.36 ± 1.92	**0.002**	0.938
In the evening	188 (24.3%)	2.56 ± 1.87	136 (29.2%)		3.22 ± 1.82	0.062	**0.001**
Total	754 (97.4%)	8.96 ± 4.55	448 (96.1%)		8.78 ± 4.66	0.234	0.309

Bold: p < 0.05, N_breastfed_ = 774, N_non-breastfed_ = 466

Results are shown as number of consumers (%) and the mean ± SD among consumers. Note that bread consumption is reported as a ratio score between whole wheat, brown and white bread whereas beverage and fruit consumption is reported as the estimated frequency of consumption per week

Overall, children consumed SSB 23.3 times a week on average, with a range of 0–75. Table 2 shows that fewer breastfed children consumed SSBs at breakfast, during lunch at home and in the evening after dinner. Between breastfed and non-breastfed children, the consumption frequency among consumers differed for lunch at home (4.3 vs 4.9 times per week) and for total weekly consumption (22.5 vs. 25.0 times per week). More detailed data on the types of beverages consumed by breastfed and non-breastfed children per eating occasion can be found in Online Resources (Table S2). For example, the difference in SSB at breakfast may be due to the higher number of breastfed children who drink water and plain tea during breakfast, and due to the higher number of non-breastfed children that consume sweetened dairy drinks at breakfast.

The overall fruit consumption was comparable between the breastfed and non-breastfed group. 96.9% of children (97.4% for breastfed, 96.1% for non-breastfed) consumed fruit at some time point during the day, with an average frequency of 8.6 times per week (range 0–30.5) (Table 2). During ‘lunch at home’ and ‘lunch at school’, 21% and 15.7% of children consumed fruits. Among consumers, non-breastfed children consumed more fruit during lunch at home and in the evening than breastfed children. However, a higher percentage of breastfed children consumed fruit in the afternoon and at dinner. Since the differences in fruit consumption pattern may be related to the consumption of other foods, additional analyses were performed regarding snacking behaviour in relation to fruit consumption. This revealed that the consumption of fruit was negatively correlated with small biscuits or candy in the afternoon (Spearman r = − 0.169, p < 0.001), but positively correlated in the evening (Spearman r = 0.184, p < 0.001). More detailed data on snack consumption per eating occasion can be found in Online Resources (Table S3). For an illustration of beverage and fruit consumption frequency over the day see Online Resources (Figure S2).

### Multivariate Regression

Following up on the previous descriptive results, it was investigated how the association between breastfeeding and children’s lifestyle factors was influenced by child and parental characteristics. Linear regression controlling for child characteristics showed that breastfeeding at one month was related to lower SSB consumption frequency and a higher bread quality score at the age of 5 (Model 1, Table 3). Parental lifestyle factors and socioeconomic factors together explained more than half of these associations (Model 2/3, Table 3). Parental educational levels were the strongest contributors to the attenuation. Breastfeeding was not found to be significantly associated with total fruit consumption, sedentary time and moderate-to-vigorous physical activity.

**Table 3:** The association between breastfeeding and a) quality of the type of bread consumed, b) weekly sugar-sweetened beverage consumption, c) weekly fruit consumption, d) sedentary time and e) mean daily moderate to vigorous physical activity

Model		β	95% CI	P-value	R^2^
a) Bread quality score					
1	Breastfeeding	.167	.033; .301	**.015**	.011
2	Breastfeeding	.133	− .004; .270	.058	.023
3	Breastfeeding	.048	− .091; .187	.500	.039
b) Sugar sweetened beverage consumption					
1	Breastfeeding	− 2.156	− 3.419; − .894	**.001**	.019
2	Breastfeeding	− 1.697	− 2.977; − .417	**.009**	.047
3	Breastfeeding	− 1.030	− 2.331; .272	.121	.074
c) Fruit consumption					
1	Breastfeeding	.252	− .337; .840	0.402	.020
2	Breastfeeding	.265	− .340; .870	0.390	.021
3	Breastfeeding	.240	− .384; .864	0.451	.022
d) Sedentary time					
1	Breastfeeding	2.349	− 6.280; 10.978	0.593	.018
2	Breastfeeding	1.442	− 7.488; 10.372	0.751	.020
3	Breastfeeding	− 0.437	− 9.530; 8.655	0.925	.032
e) Moderate-to-vigorous physical activity					
1	Breastfeeding	1.064	− 2.682; 4.810	.577	.091
2	Breastfeeding	1.122	− 2.753; 4.997	.570	.094
3	Breastfeeding	1.209	− 2.750; 5.169	.549	.098

Bold: p < 0.05, N = 1064 for a/b/c, N = 744 for d/e

Model 1: Breastfeeding versus lifestyle factor, adjusted for birth weight, gestational age, ethnicity and gender

Model 2: model 1 + adjustment for parental lifestyle factors (maternal BMI, paternal BMI, maternal smoking during pregnancy)

Model 3: model 2 + adjustment for socioeconomic factors (maternal educational level, maternal age at birth, paternal educational level, household income)

Next, we examined to what extent the beneficial association between breastfeeding and BMI was related to differences in lifestyle characteristics of the child at age 5, identified above as differences in SSB consumption and type of bread (bread score). Table 4 shows that the association between breastfeeding and BMI z-score, found in the basic multivariate linear regression model, was not attenuated by adding the bread score and SSB consumption frequency to the model (Model 2). It was mostly dependent on parental lifestyle factors, with maternal BMI in particular (p < 0.001). Gender was not an effect modifier in any of the investigated associations. The results of the sensitivity analyses using an alternative classification of breastfeeding (exclusive breastfeeding vs. no breastfeeding/combined breastfeeding) can be found in the Online Resources (Table S4/S5).

**Table 4:** Results of linear regression analysis to investigate the role of sugar-sweetened beverages and type of bread consumed in the association between breastfeeding and BMI

Model		β	95% CI	P-value	R^2^
1	Breastfeeding	− .119	− .223; − .015	.**025**	0.049
2	Breastfeeding	− .120	− .225; − .016	**.024**	0.050
	SSB consumption frequency	.001	− .004; .007	.590	
	Bread score	.021	− .027; .069	.389	
3	Breastfeeding	.006	− .093; .105	.909	0.185
	SSB consumption frequency	− .002	− .007; .003	.488	
	Bread score	.034	− .010; .078	.131	
4	Breastfeeding	.012	− .090; .113	.820	0.191
	SSB consumption frequency	− .003	− .007; .002	.303	
	Bread score	.034	− .010; .079	.131	

Bold: p < 0.05, N = 1024

Model 1: Breastfeeding at one month versus BMI z-score at the age of five, adjusted for birth weight, gestational age, ethnicity and gender

Model 2: model 1 + adjustment for SSB consumption frequency and bread score

Model 3: model 2 + adjustment for parental lifestyle factors (maternal BMI, paternal BMI, maternal smoking during pregnancy)

Model 4: model 3 + adjustment for socioeconomic factors (maternal educational level, maternal age at birth, paternal educational level, household income)

## Discussion

A number of studies has reported a potentially protective effect of breastfeeding against childhood overweight and obesity (Arenz et al. 2004; Bergmann et al. 2003; Gillman et al. 2001; Hediger et al. 2001; Liese et al. 2001; Toschke et al. 2002). In line with the previous, this study showed that BMIz at the age of five was significantly lower in breastfed compared to non-breastfed children. Furthermore, it was observed that, compared to non-breastfed children, breastfed children consumed more whole-wheat and/or brown bread relative to white bread and drink SSB less often in early life. For fruit intake, dietary patterns rather than total consumption appeared to differ between the groups. Breastfed children consume more fruit in the afternoon, which was negatively associated with candy and biscuit consumption, whereas non-breastfed children consume more fruit in the evening, which was positively associated with candy and biscuit consumption. No difference was observed in objectively measured sedentary time and moderate-to-vigorous physical activity. The association between breastfeeding and BMIz was not explained by the consumption frequency of SSB or the type of bread consumed.

The differences in dietary patterns between breastfed and non-breastfed children were small but clear. Where the vast majority of children consumed SSB, the total frequency of consumption per week was found to be approximately 10 percent lower among breastfed children. When looking at the eating pattern over the day, the difference in SSB consumption seems to be found mainly during the main meals breakfast and lunch at home, and in the evening. Based on the Dutch food composition table (NEVO Table 2016), it is estimated that the substitution of three 150 ml portions of the frequently consumed sweetened dairy drinks with water or tea without sugar during the main meals could already decrease daily caloric intake with 256 kcal. Hypothetically, this would be about 17% of the average intake of around 1500 kcal per day in this population (Dutch National Food Consumption Survey, 2007), meaning that reducing SSB consumption during main meals may considerably alter energy balance. SSB consumption during main meals may therefore be a suitable target for future dietary interventions.

In addition to lower SSB consumption, indicators of a slightly healthier dietary pattern among breastfed children included the consumption of healthier types of bread, and a larger proportion of children consuming fruit in the afternoon and with dinner. Previous studies in Australian children aged 2–8 years (Grieger et al. 2011), Dutch children aged 7–8 (Scholtens et al. 2008) and young Dutch adults aged 18–28 (De Kroon et al. 2011), have also reported healthier dietary patterns later in life in individuals who were breastfed during infancy. Although our study results are in the same direction, comparability of the studies is diminished due to differences between studies with regard to the dietary assessment methods used. The previous studies used common dietary assessment methods like 24 h recall and food frequency questionnaires, which differ from our food pattern questionnaire, in which timing of consumption is taken into account as well.

In line with previous research, we confirmed that breastfeeding was associated with lower BMI-z scores. Altough it was hypothesized that differences in dietary patterns between breastfed and non-breastfed children contributed to the difference in BMI between those groups, SSB consumption and type of bread did not explain the association between breastfeeding and BMI in this study. Similar findings were reported by previously mentioned Dutch studies, in which healthy diet characteristics did not explain the reported association between breastfeeding and lower overweight risk at the age of 8 (Scholtens et al. 2008), or the inverse association between breastfeeding duration and BMI, waist circumference and waist-hip ratio in 18–28 year olds (De Kroon et al. 2011). Together, these findings illustrate in a broad age range that the protective effect of breastfeeding against overweight does not rely on healthier dietary characteristics observed among individuals who were breastfed in infancy.

Where SSB consumption and type of bread did not explain the association between breastfeeding and BMI, factors that did strongly contribute to the difference in BMI among breastfed and non-breastfed children at the age of five were parental lifestyle factors and socioeconomic factors. This study could therefore not replicate the findings of previous studies, in which the association between breastfeeding and BMI remained significant after adjusting for these potential confounders (Bergmann et al. 2003; Gillman et al. 2001; Hediger et al. 2001; Toschke et al. 2002). That these studies did find significant associations after adjustment may be related to larger sample sizes of the studies and differences in the main outcome measure, which was overweight/obesity prevalence or incidence in the previous studies, whereas we studied BMIz.

In the Netherlands, 80% of mothers initiate breastfeeding, but this number rapidly decreases in the first weeks after birth (Peeters et al. 2015). Because of our interest in parental motivation and persistence, we made the distinction between breastfed and non-breastfed at the age of one month. Due to the dose dependent character of the association between breastfeeding and childhood obesity (Harder et al. 2005), making the distinction at this early age could have attenuated the results. However, replicating the analyses with groups based on breastfeeding status at three months (46.1% of children breastfed) led to comparable results.

The strong attenuation of the association between breastfeeding and dietary factors by parental education level, can be understood when realising that both breastfeeding and a child’s dietary pattern at age five are largely dependent on parental choices. Parents with higher education levels may adhere to a healthier dietary pattern themselves (Vinke et al. 2018), have higher control over children’s eating behaviours (Saxton et al. 2009), and have higher health literacy (Rudd et al. 2004). A higher parental health literacy, referring to the skills needed to obtain, process and understand basic health information and services, is needed to make appropriate health decisions (Berkman et al. 2011). It has previously been reported that parents with low health literacy skills are less likely to breastfeed their child (Fredrickson et al. 1995; Kaufman et al. 2001). In addition, caregivers with low health literacy are found to be less likely to look at nutrition labels on food products, and are twice as likely to have an inaccurate perception of their child’s weight (Sanders et al. 2009). Together, this makes it plausible that health literacy is involved in the association between breastfeeding and a toddler’s dietary pattern, emphasizing the importance of providing parents with easy readable or perhaps tailored child health information. As health literacy is strongly associated with education level (Rudd et al. 2004), the results indicating that parental education strongly attenuates the association between breastfeeding and SSB consumption or bread score are in line with this reasoning.

Finally, sedentary time and moderate-to-vigorous physical activity levels did not differ between breastfed and non-breastfed children. The levels of PA reported in this study are comparable to results from previous studies (Nader et al. 2008; Troiano et al. 2008). However, breastfeeding was not a determinant of PA levels at age 5 and lower PA did not necessarily cluster with unhealthier eating behaviour, suggesting that the determinants of these two lifestyle components differ.

Strengths of this study are the availability of objectively measured physical activity, and the use of a food pattern questionnaire, which provides valuable insight into the behavioural aspect of nutrition, in addition to the consumption aspect. Therefore, results are directly applicable to daily life. However, a limitation is that this food pattern questionnaire has not yet been validated, and that the questionnaire did not specify portion sizes. Furthermore, in this study we categorized children as breastfed or non-breastfed at the age of 1 month. As heterogeneity exists in the field regarding the age at which the distinction in breastfeeding is made, study results may be less comparable. Finally, this study was performed in a cohort of Dutch children. As the Netherlands is a highly developed country, results may not be generalizable.

Altogether, breastfeeding during infancy may indicate a healthy start of life which is more likely to be followed by a favorable dietary pattern at preschool age. However, differences in dietary characteristics did not explain the protective effect of breastfeeding against overweight. At the age of five, breastfed and non-breastfed children’s lifestyle most consistently differed with regard to SSB consumption frequency, typically at the main meals. In addition, breastfed children appear to be characterized by consumption of healthier types of bread and a replacement of small biscuits and candy with fruit in the afternoon. Since dietary behaviours in early childhood are largely dependent on parental choices, nutrition education for parents may be key to improve dietary patterns in children. Furthermore, the cultural acceptability of the recommendation to limit the consumption of SSB could be increased by emphasizing on limiting the consumption during main meals.

## Electronic supplementary material

Below is the link to the electronic supplementary material.

Supplementary file1 (docx 255 KB)
