# Supplementary material for: The Role of Children’s Dietary Pattern and Physical Activity in the Association Between Breastfeeding and BMI at Age 5: The GECKO Drenthe Cohort
Source: Matern Child Health J. 2020 Nov 30;25(2):338–48. doi: 10.1007/s10995-020-03063-6 (PMC7870607; doi:10.1007/s10995-020-03063-6)
Supplement: Supplementary file 1 — Supplementary file1 (docx 255 KB) [file 10995_2020_3063_MOESM1_ESM.docx]

# Supplementary materials

**The Role of Children’s Dietary Pattern and Physical Activity in the Association Between Breastfeeding and BMI at Age 5: The GECKO Drenthe Cohort**

*Maternal and Child Health Journal*

Petra Corianne Vinke, MSc.^1^, Carolien Tigelaar, BSc.^1^, Leanne Karen Küpers, PhD^1,2^, Eva Corpeleijn, PhD^1

1^ Department of Epidemiology, University Medical Center Groningen, University of Groningen, P.O. Box 30 001, 9700 RB Groningen, The Netherlands

^2^ Division of Human Nutrition and Health, Wageningen University & Research, PO Box 8129, 6700, EV, Wageningen, The Netherlands

**Corresponding Author:**

Petra Vinke
Department of Epidemiology (FA40), University Medical Center Groningen
P.O. Box 30 001, 9700 RB Groningen, The Netherlands
p.c.vinke@umcg.nl
+31(0)50 – 361058

### Figure S1: Flowchart inclusion.


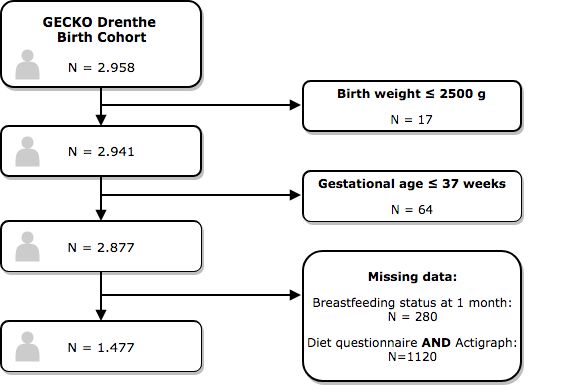


### Table S1: Type of bread

|  |  | | **Breastfed** | |  | **Non-breastfed** | |  |  |
| --- | --- | --- | --- | --- | --- | --- | --- | --- | --- |
|  |  | **N_consumer_**  **(%)** | | **Consumer intake** | | **N_consumer_**  **(%)** | **Consumer intake** | **P-value** *Consumer percentage* | **P-value** *Consumer intake* |
| Breakfast | | | | | | | | |  |
|  | White bread  Brown bread  Whole-wheat bread  White bread with fibre  Croissants  Bread rolls  Rusk bread  Other | 120 (15.5%)  561 (72.5%)  345 (44.6%)  56 (7.2%)  187 (24.2%)  316 (40.8%)  230 (29.7%)  104 (13.4%) | | 2.08 ± 1.94  5.14 ± 1.76  4.31 ± 2.16  2.25 ± 2.13  0.64 ± 0.62  0.87 ± 0.84  1.34 ± 1.41  1.98 ± 2.18 | | 102 (21.9%)  326 (70%)  176 (37.8%)  21 (4.5%)  104 (22.3%)  178 (38.2%)  136 (29.2%)  50 (10.7%) | 2.28 ± 2.12  5.18 ± 1.73  4.50 ± 2.23  2.31 ± 2.18  0.64 ± 0.58  0.90 ± 0.83  1.65 ± 1.64  1.50 ± 1.68 | **0.006**  0.363  **0.021**  0.068  0.489  0.370  0.898  0.182 | 0.584  0.747  0.248  0.897  0.974  0.599  0.092  0.342 |
| Lunch at school | | | | | | | | |  |
|  | White bread  Brown bread  Whole-wheat bread  White bread with fibre  Croissants  Bread rolls  Rusk bread  Other | 54 (7.0%)  345 (44.6%)  220 (28.4%)  27 (3.5%)  24 (3.1%)  90 (11.6%)  36 (4.7%)  31 (4.0%) | | 1.02 ± 1.04  1.76 ± 1.09  1.79 ± 1.23  1.32 ± 1.15  0.67 ± 0.56  0.79 ± 0.71  0.94 ± 0.84  1.34 ± 1.24 | | 34 (7.3%)  188 (40.3%)  104 (22.3%)  9 (1.9%)  24 (5.2%)  54 (11.6%)  16 (3.4%)  8 (1.7%) | 0.85 ± 0.92  1.86 ± 1.10  1.71 ± 1.13  0.94 ± 0.88  0.67 ± 0.56  0.83 ± 0.85  0.50 ± 0.00  1.25 ± 1.04 | 0.820  0.155  **0.019**  0.120  0.093  1.000  0.380  **0.028** | 0.396  0.338  0.687  0.398  1.000  0.917  **0.042**  0.984 |
| Lunch at home | | | | | | | | |  |
|  | White bread  Brown bread  Whole-wheat bread  White bread with fibre  Croissants  Bread rolls  Rusk bread  Other | 129 (16.7%)  548 (70.8%)  335 (43.3%)  46 (5.9%)  98 (12.7%)  259 (33.5%)  176 (22.7%)  88 (11.4%) | | 1.68 ± 1.67  4.32± 1.71  3.95 ± 1.85  1.98 ± 1.81  0.64 ± 0.52  1.04 ± 0.96  1.52 ± 1.39  2.32 ± 1.98 | | 95 (20.4%)  331 (71.0%)  183 (39.3%)  23 (4.9%)  73 (15.7%)  137 (29.4%)  102 (21.9%)  32 (6.9%) | 2.23 ± 1.99  4.50 ± 1.66  3.98 ± 1.96  1.63 ± 1.69  0.66 ± 0.65  1.10 ± 1.04  1.85 ± 1.49  2.63 ± 2.21 | 0.109  0.949  0.172  0.523  0.148  0.148  0.779  **0.010** | **0.036**  0.136  0.805  0.429  0.961  0.636  0.052  0.533 |
| Dinner | | | | | | | | |  |
|  | White bread  Brown bread  Whole-wheat bread  White bread with fibre  Croissants  Bread rolls  Rusk bread  Other | 15 (1.9%)  60 (7.8%)  37 (4.8%)  7 (0.9%)  6 (0.8%)  25 (3.2%)  14 (1.8%)  7 (0.9%) | | 2.10 ± 2.16  2.60 ± 2.22  1.96 ± 2.14  0.79 ± 0.76  0.83 ± 0.82  0.66 ± 0.55  1.21 ± 1.27  0.79 ± 0.76 | | 12 (2.6%)  40 (8.6%)  23 (4.9%)  3 (0.6%)  9 (1.9%)  19 (4.1%)  7 (1.5%)  4 (0.9%) | 2.00 ± 2.43  2.45 ± 2.24  1.46 ± 1.58  1.17 ± 1.15  0.50 ± 0.00  0.71 ± 0.63  0.79 ± 0.76  1.00 ± 1.00 | 0.547  0.592  0.892  0.751  0.104  0.433  0.822  1.000 | 0.679  0.681  0.494  0.513  0.221  0.775  0.451  0.673 |
| **Bold**: p<0.05.  Consumer percentages and consumer means based on dietary information from 774 breastfed children and 466 non-breastfed children. Data presented as number of consumers and percentage of group, plus consumer means ± standard deviation. The consumer mean represents the average frequency of consumption per week. | | | | | | | | | |

### Table S2: Type of drinks

|  |  | **Breastfed** | | **Non-breastfed** | |  |  |
| --- | --- | --- | --- | --- | --- | --- | --- |
|  |  | **N_consumer_**  **(%)** | **Consumer intake** | **N_consumer_**  **(%)** | **Consumer intake** | **P-value** *Consumer percentage* | **P-value**  *Consumer*  *intake* |
| Breakfast | | | | | | |  |
|  | Nothing  Water  Milk  Sweetened dairy drinks  Soda  Diet soda  Juice  Lemonade  Tea without sugar  Tea with sugar  Tea with milk and sugar | 22 (2.8%)  98 (12.7%)  447 (57.8%)  318 (41.1%)  8 (1.0%)  3 (0.4%)  137(17.7%)  161 (20.8%)  114 (14.7%)  69 (8.9%)  38 (4.9%) | 3.86 ± 2.34  2.93 ± 2.27  4.99 ± 1.91  3.66 ± 2.18  2.25 ± 1.98  1.83 ± 1.15  2.91 ± 2.25  3.42 ± 2.17  3.61 ± 2.48  2.94 ± 2.19  3.71 ± 2.48 | 19 (4.1%)  39 (8.4%)  239 (51.3%)  244 (52.4%)  3 (0.6%)  0 (0.0%)  69 (14.8%)  88 (18.9%)  48 (10.3%)  52 (11.2%)  25 (5.4%) | 3.87 ± 2.11  2.40 ± 2.20  4.76 ± 1.92  4.15 ± 2.25  1.83 ± 1.15  -  2.56 ± 2.20  3.64 ± 2.33  3.67 ± 2.40  2.96 ± 2.12  3.22 ± 2.44 | 0.254  **0.019**  **0.029**  **<0.001**  0.550  0.296  0.208  0.422  **0.029**  0.200  0.790 | 0.989  0.190  0.082  **0.010**  0.909  -  0.255  0.481  0.879  0.902  0.447 |
| In the morning | | | | | | |  |
|  | Nothing  Water  Milk  Sweetened dairy drinks  Soda  Diet soda  Juice  Lemonade  Tea without sugar  Tea with sugar  Tea with milk and sugar | 9 (1.2%)  94 (12.1%)  59 (7.6%)  84 (10.9%)  18 (2.3%)  6 (0.8%)  195 (25.2%)  643 (83.1%)  29 (3.7%)  14(1.8%)  7 (0.9%) | 1.61 ± 2.03  2.76 ± 1.79  3.25 ± 1.82  2.88 ± 1.90  3.50 ± 1.72  2.50 ± 2.19  3.32 ± 1.82  5.22 ± 1.64  2.22 ± 1.83  2.93 ± 2.24  1.93 ± 0.98 | 5 (1.1%)  51 (10.9%)  25 (5.4%)  61 (13.1%)  7 (1.5%)  6 (1.3%)  152 (32.6%)  368 (79.0%)  11 (2.4%)  11 (2.4%)  3 (0.6%) | 1.70 ± 1.79  2.97 ± 1.94  3.38 ± 1.74  3.29 ± 1.76  3.79 ± 1.38  0.83 ± 0.82  3.76 ± 1.82  4.88 ± 1.72  1.23 ± 1.35  1.96 ± 1.29  3.17 ± 3.06 | 1.000  0.584  0.131  0.237  0.406  0.383  **0.005**  0.082  0.245  0.535  0.751 | 0.815  0.575  0.760  0.207  0.330  0.083  **0.037**  **0.001**  0.089  0.329  0.602 |
| Lunch at school | | | | | | |  |
|  | Nothing  Water  Milk  Sweetened dairy drinks  Soda  Diet soda  Juice  Lemonade  Tea without sugar  Tea with sugar  Tea with milk and sugar | 2 (0.3%)  42 (5.4%)  210 (27.1%)  207 (26.7%)  6 (0.8%)  1 (0.1%)  89 (11.5%)  126 (16.3%)  22 (2.8%)  10 (1.3%)  4 (0.5%) | 1.50 ± 1.41  1.36 ± 1.09  1.73 ± 1.12  1.57 ± 1.09  1.17 ± 1.03  2.50  1.38 ± 1.13  1.58 ± 1.18  1.68 ± 1.47  1.30 ± 1.03  1.50 ± 1.15 | 3 (0.6%)  17 (3.6%)  96 (20.6%)  150 (32.2%)  6 (1.3%)  0 (0.0%)  43 (9.2%)  76 (16.3%)  1 (0.2%)  8 (1.7%)  5 (1.1%) | 0.50 ± 0.00  1.32 ± 1.24  1.67 ± 1.22  1.65 ± 1.12  1.83 ± 1.03  -  1.29 ± 0.99  1.42 ± 1.15  0.50  1.50 ± 1.07  0.50 ± 0.00 | 0.370  0.170  **0.010**  **0.045**  0.383  1.000  0.218  1.000  **<0.001**  0.626  0.309 | 0.221  0.799  0.533  0.557  0.269  -  0.807  0.332  0.397  0.680  0.091 |
| Lunch at home | | | | | | |  |
|  | Nothing  Water  Milk  Sweetened dairy drinks  Soda  Diet soda  Juice  Lemonade  Tea without sugar  Tea with sugar  Tea with milk and sugar | 14 (1.8%)  109 (14.1%)  476 (61.5%)  396 (51.2%)  15 (1.9%)  6 (0.8%)  112 (14.5%)  209 (27.0%)  54 (7.0%)  46 (5.9%)  18 (2.3%) | 4.21 ± 2.20  2.61 ± 1.84  4.17 ± 1.78  3.09 ± 1.86  2.90 ± 2.16  3.17 ± 2.07  2.38 ± 1.66  3.14 ± 1.81  2.46 ± 1.80  2.54 ± 1.71  2.72 ± 1.93 | 6 (1.3%)  59 (12.7%)  245 (52.6%)  273 (58.6%)  4 (0.9%)  5 (1.1%)  74 (15.9%)  126 (27.0%)  28 (6.0%)  38 (8.2%)  20 (4.3%) | 1.50 ± 1.67  2.60 ± 1.91  4.00 ± 1.89  3.64 ± 1.91  3.00 ± 1.00  2.50 ± 1.41  2.42 ± 1.64  3.52 ± 1.85  2.43 ± 1.92  2.87 ± 1.60  2.80 ± 1.87 | 0.643  0.494  **0.002**  **0.011**  0.157  0.756  0.512  1.000  0.556  0.161  0.061 | **0.019**  0.989  0.346  **<0.001**  0.663  0.617  0.794  0.058  0.881  0.302  0.864 |
| In the afternoon | | | | | | |  |
|  | Nothing  Water  Milk  Sweetened dairy drinks  Soda  Diet soda  Juice  Lemonade  Tea without sugar  Tea with sugar  Tea with milk and sugar | 8 (1.0%)  203 (26.2%)  47 (6.1%)  50 (6.5%)  55 (7.1%)  17 (2.2%)  210 (27.1%)  714 (92.2%)  112 (14.5%)  78 (10.1%)  34 (4.4%) | 1.75 ± 1.49  2.94 ± 1.85  2.25 ± 1.65  1.62 ± 1.29  2.25 ± 1.89  1.56 ± 1.60  2.73 ± 1.77  5.07 ± 1.60  2.46 ± 1.76  2.47 ± 1.49  2.62 ± 1.84 | 3 (0.6%)  100 (21.5%)  25 (5.4%)  34 (7.3%)  23 (4.9%)  14 (3.0%)  164 (35.2%)  422 (90.6%)  48 (10.3%)  53 (11.4%)  27 (5.8%) | 0.50 ± 0.00  2.96 ± 2.09  2.42 ± 1.68  2.91 ± 2.13  2.59 ± 1.76  2.07 ± 0.85  3.09 ± 1.72  4.91 ± 1.65  3.04 ± 2.01  2.46 ± 1.69  3.32 ± 1.94 | 0.550  0.065  0.707  0.562  0.147  0.453  **0.003**  0.341  **0.036**  0.505  0.280 | 0.151  0.764  0.681  **0.005**  0.297  0.124  **0.047**  0.105  0.075  0.839  0.147 |
| Dinner | | | | | | |  |
|  | Nothing  Water  Milk  Sweetened dairy drinks  Soda  Diet soda  Juice  Lemonade  Tea without sugar  Tea with sugar  Tea with milk and sugar | 259 (33.5%)  399 (51.6%)  106 (13.7%)  45 (5.8%)  32 (4.1%)  13 (1.7%)  69 (8.9%)  264 (34.1%)  9 (1.2%)  3 (0.4%)  1 (0.1%) | 4.71 ± 1.87  3.51 ± 2.20  3.26 ± 2.35  2.46 ± 2.19  1.88 ± 2.00  2.19 ± 1.80  2.56 ± 1.81  3.64 ± 2.11  1.17 ± 1.00  1.83 ± 1.15  2.50 | 177 (38.0%)  188 (40.3%)  68 (14.6%)  48 (10.3%)  19 (4.1%)  10 (2.1%)  53 (11.4%)  141 (30.3%)  1 (0.2%)  6 (1.3%)  2 (0.4%) | 4.76 ± 1.97  3.47 ± 2.18  3.56 ± 2.28  2.71 ± 1.76  2.29 ± 2.30  1.70 ± 1.40  2.76 ± 2.11  3.59 ± 2.02  2.50  1.50 ± 1.10  2.50 ± 0.00 | 0.111  **<0.001**  0.673  **0.005**  1.000  0.665  0.169  0.169  0.101  0.088  0.560 | 0.600  0.834  0.371  0.254  0.550  0.546  0.731  0.842  0.221  0.655  1.000 |
| In the evening | | | | | | |  |
|  | Nothing  Water  Milk  Sweetened dairy drinks  Soda  Diet soda  Juice  Lemonade  Tea without sugar  Tea with sugar  Tea with milk and sugar | 296 (38.2%)  342 (44.2%)  83 (10.7%)  37 (4.8%)  43 (5.6%)  18 (2.3%)  92 (11.9%)  280 (36.2%)  33 (4.3%)  24 (3.1%)  12 (1.6%) | 5.18 ± 1.78  3.42 ± 2.10  3.68 ± 2.16  2.93 ± 2.17  2.04 ± 1.74  1.61 ± 1.84  2.35 ± 1.95  3.31 ± 2.11  1.96 ± 1.68  2.00 ± 1.59  4.00 ± 1.93 | 144 (30.9%)  185 (39.7%)  42 (9.0%)  38 (8.2%)  25 (5.4%)  13 (2.8%)  72 (15.5%)  207 (44.4%)  11 (2.4%)  19 (4.1%)  10 (2.1%) | 4.79 ± 1.92  3.24 ± 2.06  3.60 ± 2.22  3.34 ± 2.21  1.86 ± 2.43  2.04 ± 1.20  2.97 ± 2.03  3.58 ± 2.12  3.05 ± 2.54  2.29 ± 1.32  4.30 ± 1.99 | **0.010**  0.124  0.381  **0.019**  1.000  0.708  0.083  **0.005**  0.083  0.423  0.507 | **0.027**  0.333  0.822  0.410  0.146  0.170  **0.032**  0.161  0.237  0.366  0.679 |
|  | **Bold**: p<0.05  Consumer percentages and consumer means based on dietary information from 774 breastfed children and 466 non-breastfed children. Data presented as number of consumers and percentage of group, plus consumer means ± standard deviation. The consumer mean represents the average frequency consumption per week | | | | | | |

### Table S3: Snacks

|  | | **Breastfed** | |  | **Non-breastfed** | | |  | |  |
| --- | --- | --- | --- | --- | --- | --- | --- | --- | --- | --- |
|  | | **N_consumer_**  **(%)** | **Consumer intake** | | **N_consumer_**  **(%)** | **Consumer intake** | **P-value** *Consumer percentage* | | **P-value** *Consumer intake* |  |
| Snacks in the morning | | | | | | | | |  |  |
|  | Nothing  Fruit  Bread  Raisins  Raw vegetables  Salty snack  Small biscuits or candy  Packed biscuits  Large cookies or cake  Gingerbread  Dairy products  Neutral snack* | 6 (0.8%)  721 (93.2%)  120 (15.5%)  32 (4.1%)  131 (16.9%)  12 (1.6%)  250 (32.3%)  258 (33.3%)  20 (2.6%)  199 (25.7%)  17 (2.2%)  80 (10.3%) | 1.83 ± 1.63  4.87 ± 1.44  2.73 ± 1.86  0.88 ± 0.94  1.78 ± 1.27  1.17 ± 1.56  2.21 ± 1.65  2.20 ± 1.61  1.10 ± 0.94  1.76 ± 1.37  2.27 ± 1.86  2.00 ± 1.54 | | 7 (1.5%)  422 (90.6%)  67 (14.4%)  14 (3.0%)  63 (13.5 %)  7 (1.5%)  136 (29.2%)  159 (34.1%)  15 (3.2%)  137 (29.4%)  9 (1.9%)  30 (6.4%) | 1.07 ± 0.98  4.73 ± 1.47  2.65 ± 2.04  1.50 ± 1.52  1.87 ± 1.38  0.79 ± 0.76  2.32 ± 1.48  2.29 ± 1.53  0.77 ± 0.70  1.76 ± 1.37  2.28 ± 2.11  1.90 ± 1.59 | 0.256  0.103  0.624  0.354  0.125  1.000  0.255  0.804  0.596  0.166  0.840  **0.023** | | 0.362  0.091  0.657  0.115  0.787  0.790  0.346  0.511  0.252  0.985  0.954  0.704 |  |
| Snacks in the afternoon | | | | | | | | |  |  |
|  | Nothing  Fruit  Bread  Raisins  Raw vegetables  Salty snack  Small biscuits or candy  Packed biscuits  Large cookies or cake  Gingerbread  Dairy products  Neutral snack* | 34 (4.4%)  447 (57.8%)  98 (12.7%)  91 (11.8%)  126 (16.3%)  163 (21.1%)  699 (90.3%)  162 (20.9%)  73 (9.4%)  205 (26.5%)  19 (2.5%)  179 (23.1%) | 2.21 ± 1.85  3.19 ± 1.67  1.85 ± 1.51  1.12 ± 0.98  2.42 ± 1.69  1.25 ± 1.18  4.20 ± 1.68  2.07 ± 1.46  1.54 ± 1.49  1.79 ± 1.23  1.76 ± 1.52  2.60 ± 1.49 | | 15 (3.2%)  223 (47.9%)  60 (12.9%)  42 (9.0%)  54 (11.6%)  83 (17.8%)  411 (88.2%)  95 (20.4%)  39 (8.4%)  115 (24.7%)  9 (21.9%)  77 (16.5%) | 2.90 ± 2.03  2.99 ± 1.52  2.43 ± 1.73  1.02 ± 0.89  1.83 ± 1.40  1.34 ± 1.13  4.18 ± 1.70  2.06 ± 1.34  1.42 ± 1.11  1.98 ± 1.19  2.06 ± 1.67  2.66 ± 1.51 | 0.367  **0.001**  0.930  0.155  **0.025**  0.186  0.251  0.829  0.542  0.503  0.694  **0.006** | | 0.242  0.168  0.031  0.657  **0.036**  0.346  0.932  0.952  0.950  0.149  0.648  0.870 |  |
| Snacks in the evening | | | | | | | | |  |  |
|  | Nothing  Fruit  Bread  Raisins  Raw vegetables  Salty snack  Small biscuits or candy  Packed biscuits  Large cookies or cake  Gingerbread  Dairy products | 525 (67.8%)  188 (24.3%)  33 (4.3%)  8 (1.0 %)  23 (3.0%)  131(16.9%)  155 (20.0%)  13 (1.7%)  26 (3.4%)  19 (2.5%)  71 (9.2%) | 5.58 ± 1.58  2.56 ± 1.87  1.59 ± 1.51  1.75 ± 1.83  0.94 ± 0.84  0.99 ± 1.03  2.11 ± 1.74  1.12 ± 1.26  0.89 ± 0.80  1.13 ± 0.96  3.57 ± 2.00 | | 258 (55.4%)  136 (29.2%)  16 (3.4%)  7 (1.5%)  15 (3.2%)  77 (16.5%)  120 (25.8%)  15 (3.2%)  19 (4.1%)  19 (4.1%)  48 (10.3%) | 5.28 ± 1.66  3.22 ± 1.82  1.63 ± 1.26  0.79 ± 0.76  1.97 ± 1.77  0.89 ± 0.80  2.80 ± 2.21  1.57 ± 1.28  1.24 ± 1.37  1.55 ± 1.22  3.21 ± 2.20 | **<0.001**  0.062  0.548  0.593  0.865  0.876  **0.020**  0.112  0.533  0.126  0.551 | | **0.002**  **0.001**  0.727  0.264  **0.033**  0.746  **0.018**  0.251  0.475  0.370  0.357 |  |
|  | **Bold**: p<0.05.  Consumer percentages and consumer means based on dietary information from 774 breastfed children and 466non-breastfed children. Data presented as number of consumers and percentage of group, plus consumer means ± standard deviation. The consumer mean represents the average frequency of consumption per week.  ***** Neutral snacks include plain crackers, breadsticks, rice crackers and small, neutral bread rolls. | | | | | | | | |  |

### Figure S2: Sugar-sweetened beverage and fruit consumption over the day


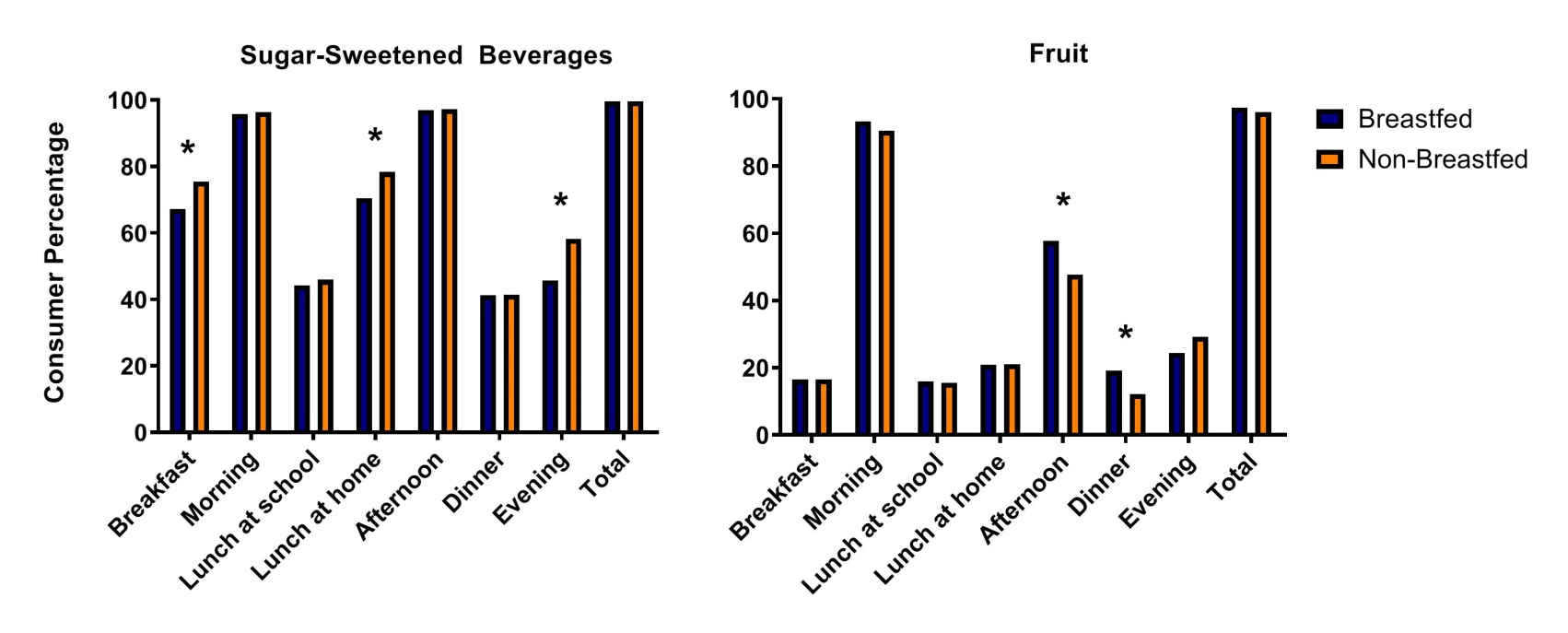


Figure S1. Percentage of children consuming sugar sweetened beverages (left) and fruit (right) at different moments of the day and over the whole day, based on dietary information from 774 breastfed children (orange) and 446 non-breastfed children (blue). *p<0.05.

| **Table S4:** The association between exclusive breastfeeding vs. no breastfeeding/combined breastfeeding and a) quality of the type of bread consumed, b) weekly sugar-sweetened beverage consumption, c) weekly fruit consumption, d) sedentary time and e) mean daily moderate to vigorous physical activity. | | | | |  |
| --- | --- | --- | --- | --- | --- |
| **Model** | | **β** | **95% CI** | **P-value** | **R^2^** |
| **a) Bread quality score** | | | | | |
| 1 | Breastfeeding | .156 | .026 ; .286 | **.019** | .011 |
| 2 | Breastfeeding | .126 | -.005; .258 | .060 | .023 |
| 3 | Breastfeeding | .047 | -.087 ; .181 | .494 | .050 |
| **b) Sugar sweetened beverage consumption** | | | | | |
| 1 | Breastfeeding | -2.360 | -3.582 ; -1.139 | **<.001** | .022 |
| 2 | Breastfeeding | -2.002 | -3.231 ; -.773 | **.001** | .050 |
| 3 | Breastfeeding | -1.332 | -2.582 ; -.081 | **.037** | .076 |
| **c) Fruit consumption** | | | | | |
| 1 | Breastfeeding | .177 | -.393 ; .747 | 0.543 | .020 |
| 2 | Breastfeeding | .180 | -.402 ; .762 | 0.544 | .021 |
| 3 | Breastfeeding | .145 | -.455 ; .746 | 0.635 | .022 |
| **d) Sedentary time** | | | | | |
| 1 | Breastfeeding | -0.492 | -8.548 ; 7.564 | 0.905 | .017 |
| 2 | Breastfeeding | -1.232 | -9.523 ; 7.059 | 0.771 | .020 |
| 3 | Breastfeeding | -3.329 | -11.759 ; 5.101 | 0.438 | .033 |
| **e) Moderate-to-vigorous Physical activity** | | | | | |
| 1 | Breastfeeding | 1.344 | -2.152 ; 4.840 | .451 | .091 |
| 2 | Breastfeeding | 1.302 | -2.295 ; 4.900 | .477 | .094 |
| 3 | Breastfeeding | 1.483 | -2.189 ; 5.155 | .428 | .099 |
| **Bold**: p<0.05, N=1064 for a/b/c, N=744 for d/e.  Model 1: Breastfeeding versus lifestyle factor, adjusted for birth weight, gestational age, ethnicity and gender. Model 2: model 1 + adjustment for parental lifestyle factors (maternal BMI, paternal BMI, maternal smoking during pregnancy)  Model 3: model 2 + adjustment for socioeconomic factors (maternal educational level, maternal age at birth, paternal educational level, household income) | | | | |  |
|  | | | | |  |
|  | | | | |  |

| **Table S5:** The association between exclusive breastfeeding vs. no breastfeeding/combined breastfeeding and BMI is not explained by sugar-sweetened beverages and type of bread consumed. | | | | | | |  |
| --- | --- | --- | --- | --- | --- | --- | --- |
| **Model** | |  |  | **β** | **95% CI** | **P-value** | **R^2^** |
| 1 |  | Breastfeeding | | -.101 | -.201 ; .000 | .**050** | 0.048 |
| 2 |  | Breastfeeding | | -.101 | -.202 ; .000 | .051 | 0.049 |
|  |  | SSB consumption frequency | | .001 | -.004 ; .007 | .607 |  |
|  |  | Bread score | | .020 | -.027 ; .068 | .406 |  |
| 3 |  | Breastfeeding | | -.008 | -.103 ; .087 | .870 | 0.185 |
|  |  | SSB consumption frequency | | -.002 | -.007 ; .003 | .477 |  |
|  |  | Bread score | | .034 | -.010 ; .079 | .128 |  |
| 4 |  | Breastfeeding | | .000 | -.097 ; .098 | .992 | 0.191 |
|  |  | SSB consumption frequency | | -.003 | -.007 ; .002 | .301 |  |
|  |  | Bread score | | .034 | -.010 ; .079 | .131 |  |
| **Bold**: p<0.05, N=1024  Model 1: Breastfeeding at one month versus BMI z-score at the age of five, adjusted for birth weight, gestational age, ethnicity and gender. Model 2: model 1 + adjustment for SSB consumption frequency and bread score  Model 3: model 2 + adjustment for parental lifestyle factors (maternal BMI, paternal BMI, maternal smoking during pregnancy)  Model 4: model 3 + adjustment for socioeconomic factors (maternal educational level, maternal age at birth, paternal educational level, household income) | | | | | | |  |
